# Critical roles of PTPN family members regulated by non-coding RNAs in tumorigenesis and immunotherapy

**DOI:** 10.3389/fonc.2022.972906

**Published:** 2022-07-26

**Authors:** Xiaolong Tang, Chumei Qi, Honghong Zhou, Yongshuo Liu

**Affiliations:** ^1^ Department of Clinical Laboratory Diagnostics, Binzhou Medical University, Binzhou, China; ^2^ Department of Clinical Laboratory, Dazhou Women and Children’s Hospital, Dazhou, China; ^3^ Key Laboratory of RNA Biology, Center for Big Data Research in Health, Institute of Biophysics, Chinese Academy of Sciences, Beijing, China; ^4^ Biomedical Pioneering Innovation Center (BIOPIC), Beijing Advanced Innovation Center for Genomics, Peking-Tsinghua Center for Life Sciences, Peking University Genome Editing Research Center, State Key Laboratory of Protein and Plant Gene Research, School of Life Sciences, Peking University, Beijing, China

**Keywords:** PTPNs, cancer, immunotherapy, miRNAs, lncRNAs, circRNAs

## Abstract

Since tyrosine phosphorylation is reversible and dynamic *in vivo*, the phosphorylation state of proteins is controlled by the opposing roles of protein tyrosine kinases (PTKs) and protein tyrosine phosphatase (PTPs), both of which perform critical roles in signal transduction. Of these, intracellular non-receptor PTPs (PTPNs), which belong to the largest class I cysteine PTP family, are essential for the regulation of a variety of biological processes, including but not limited to hematopoiesis, inflammatory response, immune system, and glucose homeostasis. Additionally, a substantial amount of PTPNs have been identified to hold crucial roles in tumorigenesis, progression, metastasis, and drug resistance, and inhibitors of PTPNs have promising applications due to striking efficacy in antitumor therapy. Hence, the aim of this review is to summarize the role played by PTPNs, including PTPN1/PTP1B, PTPN2/TC-PTP, PTPN3/PTP-H1, PTPN4/PTPMEG, PTPN6/SHP-1, PTPN9/PTPMEG2, PTPN11/SHP-2, PTPN12/PTP-PEST, PTPN13/PTPL1, PTPN14/PEZ, PTPN18/PTP-HSCF, PTPN22/LYP, and PTPN23/HD-PTP, in human cancer and immunotherapy and to comprehensively describe the molecular pathways in which they are implicated. Given the specific roles of PTPNs, identifying potential regulators of PTPNs is significant for understanding the mechanisms of antitumor therapy. Consequently, this work also provides a review on the role of non-coding RNAs (ncRNAs) in regulating PTPNs in tumorigenesis and progression, which may help us to find effective therapeutic agents for tumor therapy.

## Introduction

Protein tyrosine phosphatases (PTPs) are a wide class of enzymes that oppose protein tyrosine kinases (PTKs) (1). PTPs can be categorized into four families based on the amino acid sequence of its catalytic structural domain, each with different substrate specificities (2). Of these, 17 intracellular non-receptor PTPs, belonging to the largest class I cysteine PTP family, are designated as PTPNs (2). Extensive evidence suggests that PTPNs are involved in a range of physiological and pathological processes, including but not limited to hematopoiesis, inflammatory response, immune system, cell proliferation and differentiation, and glucose homeostasis (3–6). Furthermore, PTPNs hold a critical role in tumor progression by dephosphorylating various substrate proteins to activate or inhibit oncogenic pathways (7, 8). More notably, several PTPNs are implicated in resistance to chemotherapy and radiotherapy, and numerous studies have demonstrated that targeting certain PTPNs can boost anti-tumor immunity and efficacy, which stimulates the immune system to attack tumors (9). Therefore, PTPNs are remarkably promising therapeutic targets to combat tumors.

Non-coding RNAs (ncRNAs) are a type of RNA transcript found in many eukaryotic genomes that function as a regulator of cellular processes, including chromatin remodeling, transcription, post-transcriptional modifications and signal transduction (10, 11). In recent years, ncRNAs have been linked to the development and progression of cancer, particularly microRNAs (miRNAs), long-stranded non-coding RNAs (lncRNAs), and circular RNAs (circRNAs) (11, 12). Of these, miRNAs are defined as short ncRNAs of approximately 22 nt, lncRNAs are ncRNAs with transcripts longer than 200 nt, and circRNAs are closed continuous loop structures lacking a terminal 5’ cap and a 3’ polyadenylated tail (11). Interestingly, lncRNAs and circRNAs can perform as miRNA sponges, binding to miRNAs and altering their function. Here, ncRNAs act as tumor promoters and suppressors, depending on targeting PTPNs.

In this review, we elaborate on the roles played by PTPN family members and provide a comprehensive summary of the molecular pathways in which PTPNs are involved in various human cancers. Subsequently, the essential position occupied by PTPNs in the immune system and cancer immunotherapy is further described. Furthermore, we characterize how ncRNAs modulate PTPNs in tumorigenesis and hypothesize that ncRNA regulation in combination with immunotherapy may lead to more precise and effective efficacy.

## The physiological role of PTPNs

PTPNs, belonging to the PTP family, share the common property of possessing phosphatase activity that dephosphorylates a series of proteins, thereby governing cellular signal transduction and biological processes. Several studies have shown that PTPN2 and PTPN6 are highly expressed in hematopoietic cells and act as negative signaling regulators (13, 14). For instance, PTPN2 dephosphorylates and inactivates signal transducer and activator of transcription (STAT) protein, which is required to maintain cellular homeostasis in the hematopoietic system (15). Furthermore, PTPN2 also holds an essential role in glucose homeostasis. For example, PTPN2 negatively regulates the insulin receptor (INSR) signaling pathway through dephosphorylation of INSR and controls gluconeogenesis and hepatic glucose production through negative regulation of the interleukin-6 (IL-6) signaling pathway (16, 17). PTPN14 is required for the regulation of lymphangiogenesis (18). In addition, PTPNs perform crucial roles in the regulation of immune cell development and inflammatory responses, which will be described in later sections. Overall, the PTPN family members act as a “brake” and are essential for the maintenance of homeostasis in the body.

## Role of PTPNs in the context of cancer

**Figure 1:**
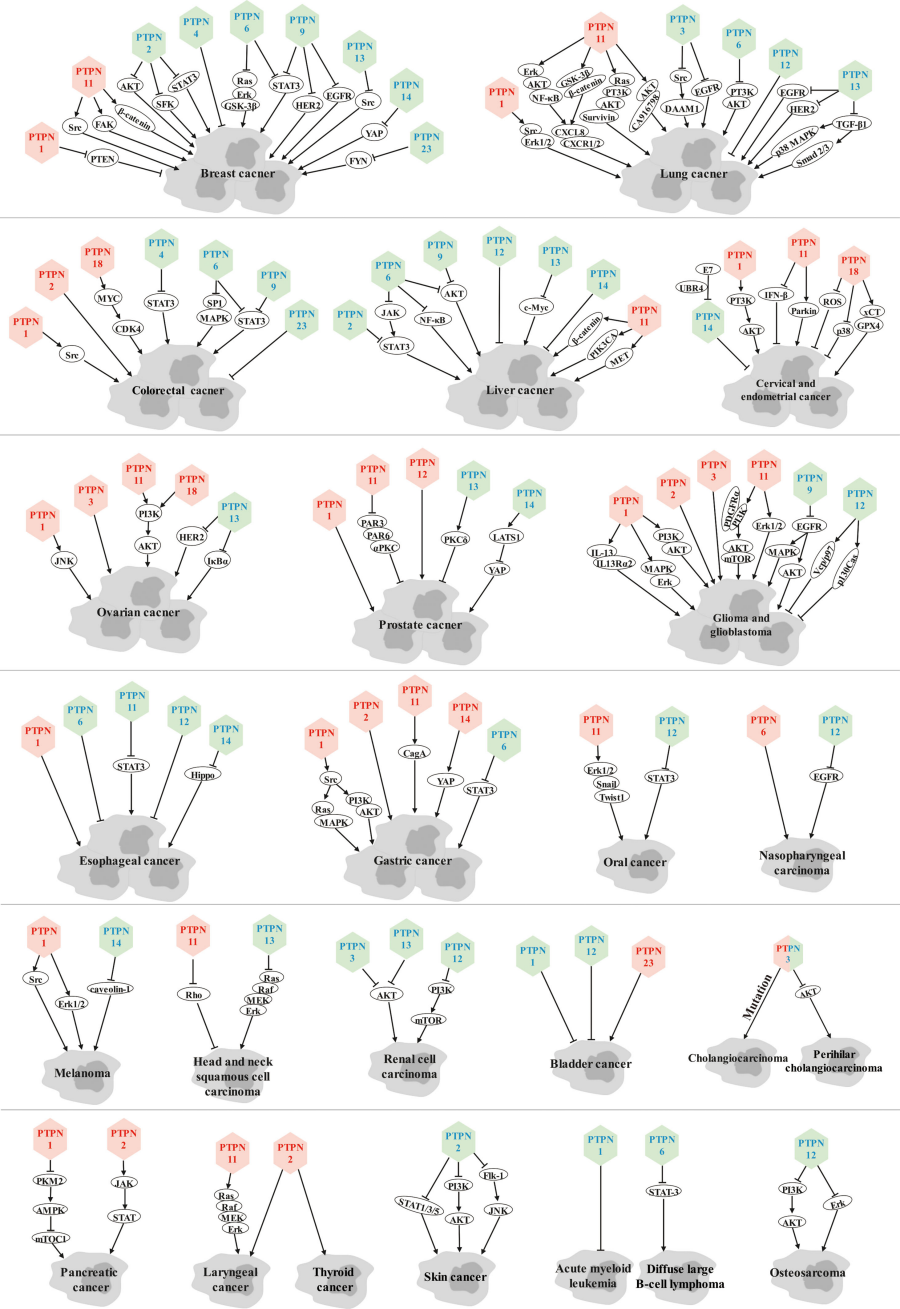
Role of PTPN family members in various cancers. Red font represents tumor promoters and blue font represents tumor suppressors.

Members of the PTPN family hold crucial roles in cancer genesis, progression, metastasis, and drug resistance by dephosphorylating a variety of substrate proteins to execute oncogenic or tumor suppressive functions in various cancers (**Figure 1**).

### Breast cancer

PTPN family members have been extensively investigated in breast cancer, with PTPN1 and PTPN11 driving the progression of breast cancer. PTPN1, which is required for invadopodia formation (19), promotes invasiveness of breast cancer cells by negatively regulating PTEN and facilitates human epidermal growth factor receptor 2 (HER2)-induced breast tumorigenesis with lung metastasis (20, 21). PTPN11 is essential for HER2, IL-6, and platelet-derived growth factor-B (PDGF-B)-induced tumorigenesis and epithelial-to-mesenchymal transition (EMT) (22–24). Mechanistically, PTPN11 enhances the oncogenic activity of β-catenin and activates Src family kinases, as well as regulates focal adhesion kinase (FAK) to promote epidermal growth factor (EGF)-induced lamellipodia persistence and migration of triple-negative breast cancer (TNBC) cells (25–27).

However, most PTPN family members function as tumor suppressors in breast cancer, including PTPN2, PTPN4, PTPN6, PTPN9, PTPN13, PTPN14, and PTPN23. Specifically, PTPN2 is implicated in the subtype specificity of breast cancer, and low expression in patients with Luminal A and HER2-positive tumors is linked to a higher recurrence rate, but not in patients with triple-negative tumors (28). More importantly, loss of PTPN2 is coupled with activation of oncogenic signaling pathways, such as protein kinase B (AKT), Src family kinase (SFK) and STAT3 signaling pathways, and also with resistance to tamoxifen (29, 30). PTPN6 dampens the oncogenic characteristics of breast cancer by dephosphorylating STAT3 and inactivating the Ras/extracellular signal-regulated kinase (Erk)/glycogen synthase kinase-3beta (GSK-3β) signaling pathway (31, 32). PTPN9 suppresses the growth and invasion of breast cancer cells by negatively regulating HER2 and epidermal growth factor receptor (EGFR) and suppressing STAT3 activation (33, 34). Furthermore, PTPN4 and PTPN13 are favorable prognostic biomarkers for breast cancer patients (35, 36), in which PTPN13 induces apoptosis of breast cancer cells and inhibits breast tumor aggressiveness by directly inactivating Src kinase and stabilizing intercellular adhesion and promoting desmosome formation (37–39). PTPN14 negatively regulates the oncogenic function of yes-associated protein (YAP) by modulating the subcellular localization of YAP and suppressing its transcriptional co-activator activity (40), and also inhibits breast cancer metastasis by altering protein transport (41). PTPN23 is identified as a suppressor of cell motility and invasion in breast cancer cells by inhibiting FYN kinase (42).

### Lung cancer

As with breast cancer, PTPN1 and PTPN11 also serve as oncogenic factors in lung cancer. PTPN1 contributes to the proliferation and metastasis of non-small cell lung cancer (NSCLC) cells by enhancing the Erk1/2 signaling pathway and diminishing the expression of p-Src (Tyr527), which activates Src (43). Of note, PTPN11 is not only essential for lung cancer cell growth, but also confers chemotherapy resistance (44). Mechanistically, overexpression of PTPN11 increases resistance to tyrosine kinase inhibitors (TKI) in either EGFR mutant or EGFR wild-type NSCLC cells through Erk-AKT- nuclear factor kappa B (NF-κB) and GSK3β-β-Catenin signaling pathway-mediated C-X-C motif chemokine ligand 8 (CXCL8)-chemokine receptor 1/2 (CXCR1/2) feedback loop that promotes stemness and tumorigenesis (45, 46). Moreover, PTPN11 confers cisplatin resistance to lung cancer cells through activation of the AKT-CA916798 pathway and the Ras/phosphoinositide 3-kinase (PI3K)/AKT1/survivin pathway, respectively (47, 48). Consequently, PTPN11 inhibitors in combination with chemotherapy may be a promising therapy strategy for patients with lung cancer.

Furthermore, PTPN3, PTPN6, PTPN12 and PTPN13 play tumor suppressor roles in lung cancer. PTPN3 suppresses lung cancer cell proliferation and migration by counteracting Src-mediated disheveled-associated activator of morphogenesis 1 (DAAM1) activation and actin polymerization and by promoting EGFR endocytic degradation (49, 50). Similarly, PTPN6 inactivates PI3K/AKT signaling pathway (51). PTPN12 is a valuable prognostic biomarker for patients with NSCLC (52). PTPN13 negatively regulates the growth and migration of lung cancer cells *in vitro* and inhibits tumorigenicity *in vivo* by controlling tyrosine phosphorylation of EGFR and HER2 and by inhibiting transforming growth factor beta1 (TGF-β1)-induced activation of p38 mitogen-activated protein kinase (MAPK) and Smad 2/3 pathways, respectively (53, 54).

### Colorectal cancer

In colorectal cancer, PTPN1, PTPN2 and PTPN18 undoubtedly drive its progression. Among them, PTPN1 expression is related to poor prognosis in colorectal cancer patients through dephosphorylation of the Tyr530 site of Src, which activates the Src signaling pathway and enhances the oncogenicity of colon cancer (55, 56). Similarly, high expression of PTPN2 is implicated in the incidence of colorectal cancer (57), and specific deletion of PTPN2 in bone marrow cells and macrophages prevents the development of colorectal cancer, although it promotes inflammation in the intestine (58). PTPN18 activates myelocytomatosis oncogene (MYC) signaling pathway and further potentiates cyclin-dependent kinase 4 (CDK4) expression to promote colorectal cancer progression (59).

PTPN4, PTPN6 and PTPN9, as tumor suppressors, suppress the progression of colorectal cancer by dephosphorylating pSTAT3 at the Tyr705 residue and restraining the transcriptional activity of STAT3 (60–62). Moreover, PTPN6 also facilitates chemosensitivity of colorectal cancer cells by inhibiting specificity protein 1 (SP1)/MAPK signaling pathway (63). Additionally, PTPN23 also suppresses the proliferation and EMT of human intestinal cancer cells (64).

### Liver cancer

The vast majority of the reported PTPN family members hold an inhibitory role in hepatocellular carcinoma (HCC), with the exception of PTPN11, which drives HCC progression by potentiating oncogenic proteins such as β-Catenin, PIK3CA and MET, and is associated with chemoresistance in HCC patients (65, 66).

PTPN2 can prevent hepatocyte progression to HCC by inactivating STAT3 signaling and suppressing T-cell recruitment in obese C57BL/6 mice (67). Consistently, PTPN6 overexpression inhibits the proliferation, migration, invasion and tumorigenicity of HCC cells by suppressing multiple oncogenic pathways, including janus kinase (JAK)/STAT, NF-κB and AKT signaling pathways (68). Likewise, PTPN9 contributes to the inhibition of HCC growth and metastasis by repressing the AKT pathway (69). In addition, PTPN12 and PTPN13 are associated with a favorable prognosis in HCC patients. Mechanistically, PTPN13 suppresses HCC progression by directly and competitively binding insulin-like growth factor 2 mRNA-binding protein 1 (IGF2BP1) to diminish the intracellular concentration of functional IGF2BP1, thereby promoting c-Myc mRNA degradation (70). Furthermore, PTPN14 significantly represses the proliferation, migration, and invasion of HCC cells *in vitro* and tumor growth and metastasis *in vivo* (71).

### Cervical and endometrial cancer

Currently, most PTPN family members perform oncogenic roles in cervical and endometrial cancers, with the exception of PTPN14. In detail, PTPN1 expression is linked to poor prognosis in cervical cancer possibly through activation of the PI3K/AKT pathway (72). Importantly, PTPN11 contributes to the growth and migration of cervical cancer cells and decreases the sensitivity of cells to cisplatin (73). Specifically, PTPN11 facilitates cervical cancer cell proliferation by suppressing interferon-β (IFN-β) production and restricts chemotherapeutic drug-induced apoptosis of cervical cancer cells through Parkin-dependent autophagy (74, 75). Likewise, PTPN18 promotes proliferation and metastasis and restrains apoptosis in endometrial cancer (76). Mechanistically, silencing of PTPN18 induced ferroptosis in endometrial cancer cells by increasing intracellular reactive oxygen species (ROS) levels and p-p38 expression as well as decreasing the expression of glutathione peroxidase 4 (GPX4) and system xc(-) cystine/glutamate antiporter (xCT) (72).

What’s more, the deletion of PTPN14 contributes to human papillomavirus (HPV)-mediated cervical carcinogenesis, while the major transforming activity of high-risk HPV is linked to the E7 oncoprotein. Mechanistically, the crystal structure of the terminal structural domain of E7 C binds to the catalytic structural domain of PTPN14 and induces proteasome-mediated degradation of PTPN14 *via* the ubiquitin ligase UBR4 (77, 78), thereby restricting keratin-forming cell differentiation and contributing to HPV-mediated tumorigenesis (79).

### Ovarian cancer

PTPN family members almost contribute to the progression of ovarian cancer, except for PTPN13. PTPN1 and PTPN6 are highly expressed in ovarian cancer cell lines (80, 81), in which PTPN1 accelerates ovarian cancer progression in a c-Jun N-terminal kinase (JNK)-dependent mechanism (80). Strikingly, PTPN3 confers chemoresistance and tumor stem cell-like characteristics to ovarian cancer cells (82). Furthermore, PTPN11 and PTPN18 potentiate ovarian cancer invasion and metastasis through activation of PI3K/AKT axis (83, 84).

However, high PTPN13 expression in patients with high-grade plasma ovarian cancer is related to better prognosis (85). Mechanistically, PTPN13 dephosphorylates the signaling domain of HER2 and the phosphorylation of tyrosine 42 on IκBα (IκBα-pY42), respectively, thereby attenuating the invasiveness and metastasis of ovarian cancer (86, 87).

### Prostate cancer

In prostate cancer, PTPN1 and PTPN12 are linked to poor prognosis in patients (88, 89). Importantly, PTPN11 promotes prostate cancer metastasis by attenuating the PAR3/PAR6/atypical protein kinase C (aPKC) polarity protein complex, resulting in disruption of cell polarity, dysregulation of cell-cell junctions, and increased EMT (90).

In contrast, PTPN13 and PTPN14 function as tumor suppressors in prostate cancer. Specifically, PTPN13 suppresses the proliferation and migration of prostate cancer cells and stimulates apoptosis mediated by PKCδ (91). Furthermore, PTPN14 restrains cell proliferation and invasion by enhancing phosphorylation of YAP through activation of large tumor suppressor 1 (LATS1), an effect that leads to a significant decrease in YAP-mediated transcriptional activity (92).

### Glioma and glioblastoma

PTPN1 and PTPN2 can be used as predictors of poor prognosis in glioma patients (93, 94). Mechanistically, PTPN1 promotes glioma progression through activation of MAPK/Erk and PI3K/AKT pathways as well as IL-13-mediated adhesion, migration and invasion of IL13Rα2-expressing cancer cells (93, 95). Up-regulation of PTPN2 expression induced by inflammatory response and oxidative stress contributes to glioma progression (96). Furthermore, PTPN11 regulates proliferation and tumorigenicity of glioma stem cells (97).

Likewise, PTPN2 and PTPN3 are correlated with poor patient prognosis in glioblastoma (GBM) (94, 98). In Ink4a/Arf-deficient glioblastomas, PTPN11 regulates the interaction of PI3K with PDGFRα and activates the downstream AKT/mTOR pathway, ultimately promoting tumorigenesis (99). In addition, the multivariate signaling regulatory function of PTPN11 holds a crucial role in GBM cellular decision-making. PTPN11-driven Erk1/2 activity is dominant in driving cellular proliferation and PTPN11-mediated antagonism of STAT3 phosphorylation prevails in the promotion of GBM cell death in response to EGFR and c-MET co-inhibition (100).

But for PTPN9, which appears to be a tumor suppressor, leads to decreased glioma cell viability by reducing the phosphorylation of EGFR and cooperating with BRAF (V600E) inhibitors to restrain MAPK and AKT signaling (101). Furthermore, PTPN12 controls GBM cell growth and invasion by interacting with the ATP-dependent ubiquitin segregase valosin-containing protein (Vcp)/p97 and regulating phosphorylation and stability of the focal adhesion protein p130Cas (Crk-associated substrate) (102).

### Esophageal cancer

Interestingly, PTPN1 expression is implicated in the incidence of esophageal cancer (57), while PTPN6 is down-regulated in esophageal cancer and PTPN12 is a favorable prognostic biomarker for patients with esophageal squamous cell carcinoma (103, 104). What’s more, PTPN11 and PTPN14 suppress malignant progression and chemoresistance in esophageal cancer through dephosphorylation of STAT3 and negative regulation of the Hippo signaling pathway, respectively (105, 106).

### Gastric cancer

PTPN1 significantly promotes gastric cancer (GC) cell proliferation *in vitro* and tumor growth *in vivo* by regulating Src-related signaling pathways, such as the Src/Ras/MAPK and Src/PI3K/AKT pathways (107–109). Furthermore, PTPN1 is implicated in the poor prognosis of gastric cancer, and PTPN2 is linked to the incidence of gastric cancer (57, 109). It is well known that *Helicobacter pylori* is a high risk factor for gastric cancer. Jing Jiang et al. reveals that PTPN11 expression is elevated in gastric cancer with *H. pylori* infection (110). In the early stages of gastric carcinogenesis, CagA from *H. pylori* translocates into gastric epithelial cells, undergoes tyrosine phosphorylation, and binds to PTPN11 in the human gastric mucosa *in vivo* to form a complex which is thought to contribute to the development of gastric cancer (111). SHIP2, similar to PTPN11, also binds to CagA in a tyrosine phosphorylation-dependent manner and increases CagA delivery into gastric epithelial cells (112). Of note, PTPN14 enhances the proliferation and migration of GC cells by promoting YAP phosphorylation in the Hippo signaling pathway (113). On the contrary, PTPN6 attenuates the invasion and migration of GC cells by dephosphorylating STAT3 (114).

### Other cancers

In oral cancer, PTPN11 is significantly up-regulated and promotes cell invasion and metastasis through the Erk1/2-Snail/Twist1 pathway (115). In contrast, PTPN12 suppresses oral cancer cell proliferation and invasion through induction of STAT3 dephosphorylation (116). In nasopharyngeal carcinoma (NPC), PTPN6 potentiates radioresistance and restrains cellular senescence (117). However, PTPN12, a favorable prognostic biomarker for NPC patients, suppresses the proliferation and migration of NPC cells through negative regulation of EGFR (118). In melanoma, PTPN1 promotes melanoma progression by activating the Src signaling pathway through dephosphorylating the Tyr530 site of Src as well as by enhancing the Erk1/2 signaling pathway, respectively (119, 120). Moreover, PTPN14 blocks caveolin-1-induced cancer cell metastasis by decreasing phosphorylation at the Tyr14 site of caveolin-1 (121). In the head and neck squamous cell carcinoma, PTPN11 promotes invadopodia formation through suppression of Rho signaling, leading to cancer metastasis (122). Yet, PTPN13 controls the progression of spontaneous or HPV-induced squamous cell carcinoma by inhibiting Ras/Raf/MEK/Erk signaling (123). In clear cell renal cell carcinoma, PTPN3 and PTPN13 act as tumor suppressors by inactivating the AKT signaling pathway (124, 125), whereas PTPN12 restrains the proliferation of renal cell carcinoma by inhibiting PI3K/mechanistic target of rapamycin (mTOR) pathway activity (126). In bladder cancer, PTPN1 and PTPN12 function as tumor suppressors to attenuate the growth, invasion and migration of cancer cells (127, 128). However, PTPN23 can regulate the motility of bladder cancer cells (129). In cholangiocarcinoma more than 40% of PTPN3 somatic mutations, activation of PTPN3 mutations promotes cancer cell proliferation and migration and is linked to cancer recurrence (130). Strikingly, PTPN3 suppresses proliferation through inhibition of AKT phosphorylation and is correlated with a favorable prognosis in patients with perihilar cholangiocarcinoma (131). In pancreatic cancer, PTPN1 and PTPN2 are highly expressed and associated with poor survival. Specifically, PTPN1 directly decreases pyruvate kinase M2 (PKM2) Tyr105 phosphorylation, which further leads to AMP-activated protein kinase (AMPK) inactivation, thereby increasing mTOC1 activity. PTPN2 activates the JAK-STAT signaling pathway to promote cancer progression (132, 133). Furthermore, inflammatory response and oxidative stress induce up-regulation of PTPN2, which accelerates the progression of laryngeal and thyroid cancers (134, 135). PTPN11 also promotes laryngeal cancer growth through the Ras/Raf/Mek/Erk pathway and serves as a prognostic indicator for laryngeal cancer (136). Several studies have shown that PTPN2, which exerts tumor suppressive effects in skin carcinogenesis, suppresses proliferation and induces apoptosis by negatively regulating multiple oncogenic signaling pathways, including STAT1, STAT3, STAT5, PI3K/AKT, and fetal liver kinase 1 (Flk-1)/JNK signaling pathways (137–139). In hematologic tumors, specific deficiency of PTPN1 in mouse bone marrow accelerates the development of acute myeloid leukemia (140), and PTPN6 inhibits the progression of diffuse large B-cell lymphoma by dephosphorylating STAT3 (141). Finally, PTPN12 inhibits tumor progression in osteosarcoma cells probably by inactivating PI3K/AKT and Erk pathways (142).

In summary, we have comprehensively described the role of PTPN family members in human cancers and observed that various PTPN family members are implicated in almost all oncogenic phenotypes, such as tumor proliferation, metastasis, and drug resistance, through unique molecular pathways. Interestingly, the majority of PTPN family members perform oncogenic or tumor suppressive functions depending on the tumor in which they are located. Nevertheless, a small number of PTPN family members exert more specific functions, for instance, PTPN11 as a tumor promoter whereas PTPN13 as a tumor suppressor in almost all cancers.

## Dual role of PTPNs in specific cancers

**Figure 2:**
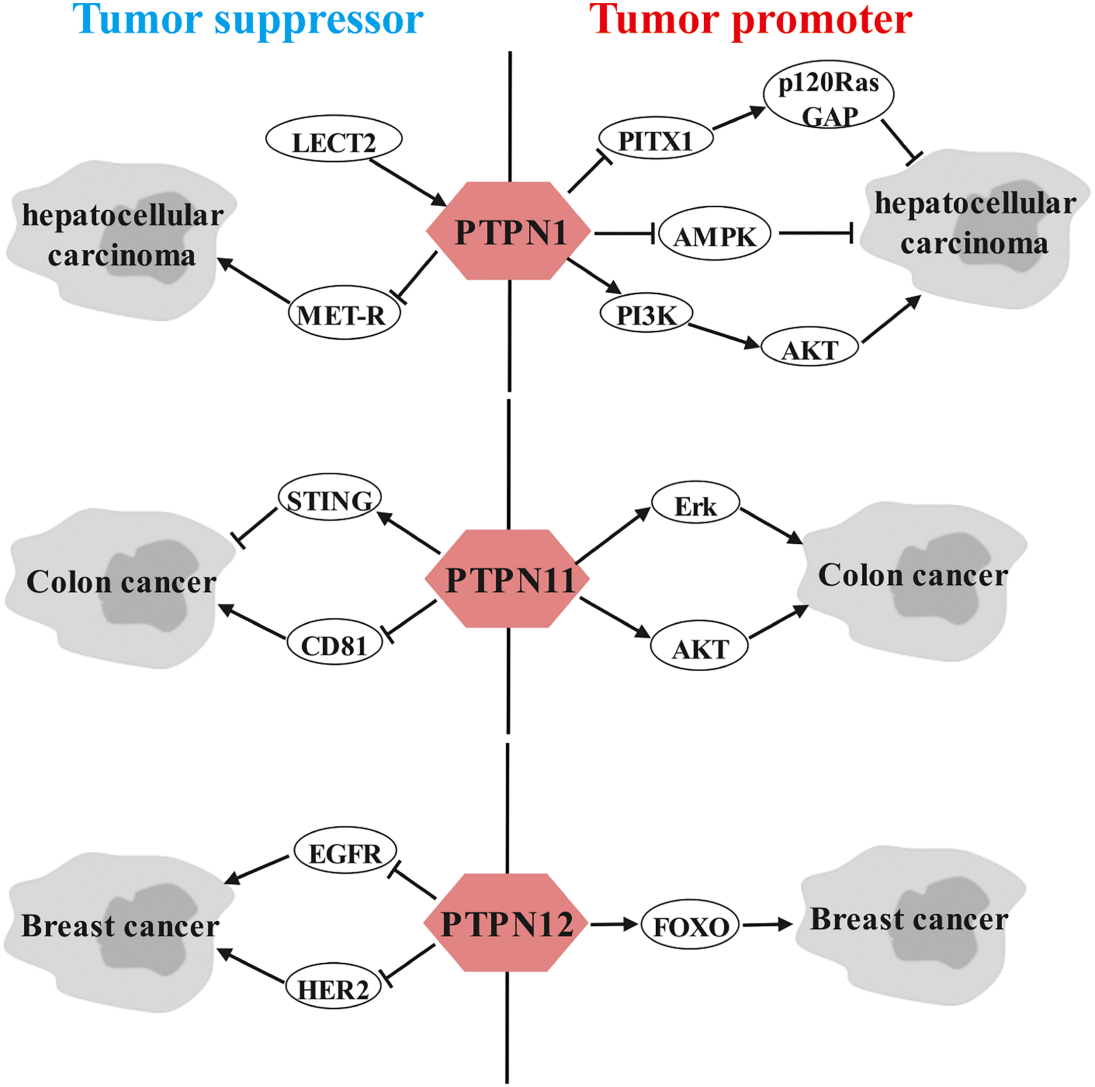
Dual role of PTPNs in specific cancers.

Strikingly, we observed that a portion of the PTPN family members have dual roles in the same cancer. For instance, PTPN1 in hepatocellular carcinoma, PTPN11 in colon cancer and PTPN12 in breast cancer can be both tumor promoters and tumor suppressors based on different molecular pathways (**Figure 2**). Elucidating the dual roles of certain PTPN may lead to better understanding of its exact functions in tumorigenesis.

### Dual-sidedness of PTPN1 in hepatocellular carcinoma

Leukocyte-derived chemotoxin 2 (LECT2), a tumor suppressor in HCC, contributes to blocking vascular invasion and metastasis in HCC by recruiting PTPN1 to antagonize MET receptor activation (143). However, Wei-Tien Tai et al. and Fang Yuan et al. proposed that PTPN1 exerts a carcinogenic role in HCC. Specifically, Pituitary homeobox 1 (PITX1) exerts a tumor suppressive effect in hepatocarcinogenesis through regulation of Ras guanosine triphosphatase-activating protein (p120RasGAP) expression levels, but PTPN1 attenuates the protein stability of PITX by directly dephosphorylating PITX1 at residues Y160, Y175 and Y179 (144). Furthermore, down-regulation of PTPN1 expression inhibits HCC progression possibly by inactivating the PI3K/AKT pathway and activating the AMPK pathway (145).

### Two faces of PTPN11 in colon cancer

Controversially, Wang Y et al. (146) and Yu M et al. (147) demonstrated that PTPN11 promotes vascular growth and proliferation of colon cancer cells as well as resistance to oxaliplatin through AKT and Erk signaling pathways. However, Yuan H et al. (148) and Wei B et al. (149) argued that PTPN11 possesses anticancer activity in colon cancer. Mechanistically, the anticancer effects of PTPN11 are achieved by interacting with CD81 and inhibiting its expression and by inhibiting DNA repair and enhancing stimulator of interferon genes (STING) pathway-mediated antitumor immunity, respectively.

### Dual role of PTPN12 in breast cancer

In addition, several studies have shown that PTPN12 is linked to a favorable prognosis in TNBC patients by suppressing multiple oncogenic tyrosine kinases, including HER2 and EGFR, thereby dampening breast cancer cell survival, migration and EMT (150–152). However, Harris IS et al. noted that PTPN12 is highly expressed in TNBC and promotes resistance to oxidative stress and supports tumorigenesis by regulating forkhead box O (FOXO) signaling (153).

Regarding the controversy of the three PTPN members mentioned above in specific cancers, we have speculated the following three reasons. First, there may be some differences in various experimental settings and tumor cell lines. Second, there are also differences in molecular mutation profiles in tumor cell lines. If mutations occur in the regulator of PTPN, it might lead to a change in the function of PTPN so that the exact opposite function appears in the same cancer. Furthermore, mutations in PTPN itself can result in opposite functions, as previously described, where PTPN3 inhibits the progression of cholangiocarcinoma by suppressing the AKT signaling pathway, whereas mutated PTPN3 promotes the oncogenic properties of cholangiocarcinoma (130, 131). Likewise, mutations occurring in PTPN11 significantly potentiate its function, producing oncogenes that facilitate the proliferation of leukemic cells (154). Third, PTPNs possess phosphatase active structural domains that regulate the dephosphorylation of many substrate proteins, including tumor promoters or suppressors, so focusing on only one molecular pathway may introduce bias for the overall role of PTPNs in a particular cancer.

Therefore, the study of PTPNs in combination with cell lines *in vitro* and mouse genetic studies *in vivo*, by knocking out specific regulators and effectors, will contribute to a better understanding of the specific signaling pathways regulated by PTPNs.

## PTPNs are crucial targets for regulating immune cells development and cancer immunotherapy

**Figure 3:**
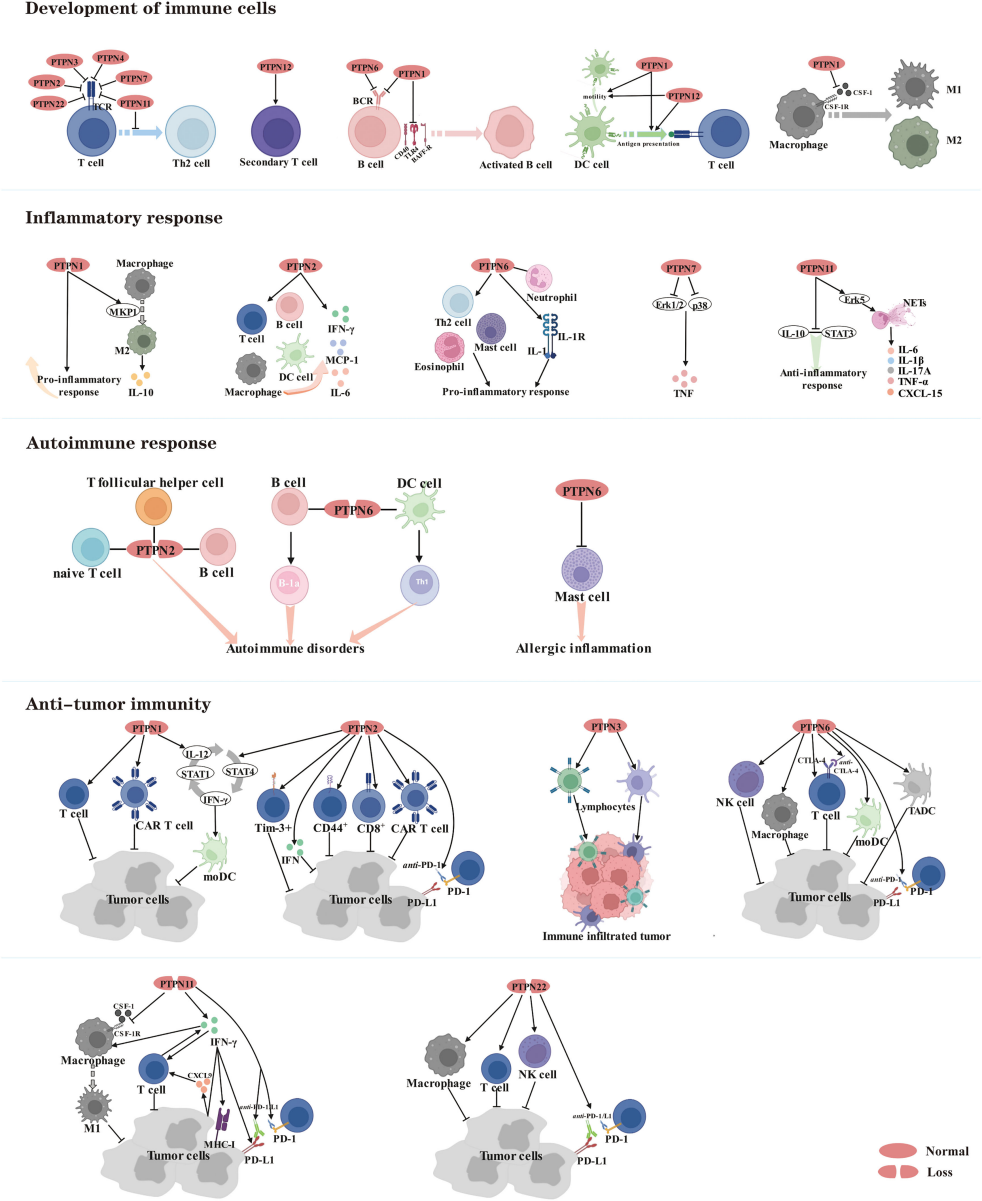
Members of the PTPN family are involved in regulating the development of immune cells and serve as therapeutic targets for inflammatory diseases, autoimmune diseases and cancer.

Given that PTPNs occupy important roles in human tumors, in addition to achieving pro- or anti-tumor effects by modulating multiple oncogenic pathways, perhaps they also reshape the tumor microenvironment by inducing tumor cells to secrete various cytokines and chemokines. It is well known that immune cells are an essential component of the tumor microenvironment (TME) (155). There is growing evidence that some PTPN family members can negatively regulate the development and differentiation of immune cells to achieve an anti-inflammatory response and prevent the onset of autoimmune diseases, but on the other hand provide an opportunity for tumors to evade surveillance by the immune system. Since the immunosuppressive tumor microenvironment creates the appropriate conditions for tumor proliferation and metastasis, an accumulating number of studies indicate that targeting PTPNs could activate the body’s immune system, thereby enhancing the efficacy of anti-tumor immunity (**Figure 3**).

### PTPNs mediate the physiological functions of immune cells

During development and maturation, various inhibitory receptors linked with phosphatases are expressed by subsets of T cells. Phosphatases, in turn, dephosphorylate key players in receptor signaling pathways. Several studies have shown that PTPN family members are crucial for suppressing T cell activation, with PTPN2, PTPN3, PTPN4, PTPN7, PTPN11 and PTPN22 negatively regulating T-cell receptor (TCR) signaling (156–163). Specifically, PTPN2 dephosphorylates and inactivates Src family kinases to regulate T cell responses (162). PTPN22 functions in a synergistic manner when forming a complex with C-terminal Src kinase (CSK), and dissociation of this complex is necessary to recruit PTPN22 to the plasma membrane, where it down-regulates TCR signaling and inhibits T cell activation (164). However, end-binding protein 1 (EB1) specifically binds to the P1 structural domain of PTPN22 by competing with CSK, which contributes to the regulation of TCR signaling (165). Another essential purpose of PTPN11 is to prevent T cells from differentiating into the T-helper 2 (Th2) phenotype (166). However, phosphatase activities of PTPN3 and PTPN4 are now dispensable for T cell development and T cell effector function (156, 157, 167). PTPN12, although not required for T cell development or primary responses, promotes secondary T cell responses by dephosphorylating the protein tyrosine kinase Pyk2 (168).

Likewise, PTPN1 and PTPN6 are also negative regulators of B-cell receptor (BCR) signaling and hold a vital role in modulating B cell activation and immunological tolerance (169, 170). Mechanistically, PTPN1 restricts B cell activation *via* negatively regulating CD40, B cell activating factor receptor (BAFF-R), and toll-like Receptor 4 (TLR4) signaling in B cells (169).

In Dendritic cells (DCs), ablation of PTPN1 and PTPN12 impairs motility *in vivo* and prevents efficient antigen presentation to T cells (171, 172). These results indicate that PTPN1 and PTPN12 hold a significant regulatory role in modulating a central DC function of initiating adaptive immune responses in response to innate immune cell activation.

Pro-inflammatory macrophages M1 and tolerance-inducible macrophages M2 are the two major subpopulations of macrophages, the former mediating host defense and the latter undertaking homeostatic and tissue regeneration functions (173). PTPN1 negatively regulates macrophage development through macrophage-colony stimulating factor 1 (CSF-1) signaling. Mechanistically, PTPN1 deficiency tends to make cells more sensitive to CSF-1, resulting in increased phosphorylation of the CSF-1 receptor (CSF-1R) in bone marrow-derived macrophages and increased inflammatory activity, implying that PTPN1 is a critical regulator of bone marrow differentiation and macrophage activation *in vivo* (174, 175).

### PTPNs perform pivotal roles in the regulation of inflammatory and autoimmune responses

Currently, several studies place PTPN family members as a double-edged sword in the regulation of inflammatory responses. PTPN1 deletion displays enhanced inflammatory activity *in vitro* and *in vivo* through constitutive overexpression of activation markers as well as greater sensitivity to endotoxins (174). However, PTPN1 deletion increases Mitogen-activated protein kinase phosphatase-1 (MKP1) expression in mouse macrophages, facilitating M2 macrophage polarization, which promotes the production of anti-inflammatory cytokine (IL-10) according to another study (176).

PTPN2 is a negative regulator of cytokine signaling, and its loss-of-function carriers have increased susceptibility to the development of inflammatory diseases (173). For instance, PTPN2^-/-^ mice develop progressive systemic inflammatory diseases with dermatitis, liver inflammation, chronic myocarditis, gastritis, nephritis, and salpingitis, as well as elevated serum interferon-γ (IFN-γ) (177). Mechanistically, PTPN2 deficiency promotes increased infiltration of B and T lymphocytes, macrophages and DCs (178, 179), and up-regulated IFN-γ induces STAT signaling and secretion of IL-6 and monocyte chemoattractant protein-1 (MCP-1) (180).

PTPN6 is a key regulatory protein in the control of inflammatory cell signaling, and its knockdown not only increases systemic inflammation in mice, but more importantly, is also implicated in human inflammatory diseases (181). For instance, Th2 cell production and mast cell-specific cytokine production are potentiated in Motheaten mice with a natural mutation in PTPN6. In an OVA-induced model of allergic airway inflammation, eosinophil inflammation, mucus hypersecretion, and airway hyperresponsiveness are enhanced in Motheaten mice, all of which contribute to the development of allergic disease (182). Furthermore, conditional deletion of PTPN6 in neutrophils is sufficient to initiate IL-1 receptor-dependent inflammatory skin diseases. Mechanistically, PTPN6 prevents caspase-8- and Ripk3-Mlkl-dependent cell death and concomitant IL-1α/β release (183).

PTPN7 exerts an anti-inflammatory function by negatively regulating Erk1/2 and p38, which enhance pro-inflammatory tumor necrosis factor (TNF)-production (184).

Intriguingly, PTPN11 holds many functions in distinct cells. PTPN11 protects mice from intestinal inflammation in epithelial cells (185), while promotes colitis and colitis-driven colon cancer in macrophages (186). Mechanistically, PTPN11 impairs IL-10-STAT3 signaling and its dependent anti-inflammatory response in human and mouse macrophages (186). Additionally, PTPN11 promotes the formation of neutrophil extracellular traps (NETs) through the Erk5 pathway, leading to pro-inflammatory cytokines such as TNF-α, IL-1β, IL-6, IL-17A, and CXCL-15, which exacerbate the inflammatory response (187).

Currently, despite extensive research on the mechanisms underlying autoimmune diseases, the fundamental causes remain unknown. Here, the main focus is on the regulatory mechanisms by which PTPN2 and PTPN6 can be involved in autoimmunity. Autoimmunity is characterized by a significant increase associated with antinuclear antibodies, inflammatory cytokines and immunoglobulins. Autoimmunity is exacerbated by PTPN2 deficits in numerous immune lineages, including naive T cells, T follicular helper cells (Tfh), and B cells (188). PTPN6 deficiency in B cells and DC cells promotes B-1a cell development and Th1 cell differentiation, respectively, leading to autoimmune disorders (189, 190). In addition, PTPN6 acts as a key negative regulator in allergic inflammation and allergen-induced anaphylaxis by regulating the function of mast cells (191).

### PTPNs are emerging targets for cancer immunotherapy

In recent years, significant progress has been made in the immunotherapy of cancer. With the intensive exploration of immune checkpoints, several PTPN family members have been revealed to possess essential roles in anti-tumor immunity and are promising therapeutic targets.

PTPN1 is elevated in intra-tumor T cells, and knocking it out promotes T cell antitumor activity and chimeric antigen receptor (CAR) T cell efficacy against solid tumors (192). Furthermore, deletion of PTPN1 and PTPN2 in DCs stimulated the growth of IL-12 and IFN-γ, which amplified the IL-12/STAT4/IFN-γ/STAT1/IL-12 positive autocrine loop, boosting the therapeutic potential of mature monocyte-derived dendritic cells (moDCs) in tumor-bearing mice (193).

In several studies, PTPN2 has been proven to be a negative regulator of interferon signaling (194, 195). Lack of PTPN2 in tumor cells enhances immunotherapy efficacy through augmenting interferon-mediated antigen presentation and growth inhibition (196). What’s more, PTPN2 deficiency in T cells boosts the generation of Tim-3^+^ cells, CD44^+^ effector/memory T cells, and CD8^+^ T cell infiltration and cytotoxicity in tumors, as well as the efficacy of *anti*- programmed cell death protein 1 (PD-1) and CAR T cells in solid tumors by promoting activation of Src family kinase LCK and cytokine-induced STAT-5 signaling (195, 197–199), which can actually facilitate tumor control and improve immunotherapy potency.

Furthermore, inhibition of PTPN3 in lymphocytes expands the proportion of tumor-infiltrating lymphocytes and activated lymphocyte cytotoxicity, as well as the anticancer effect on small cell lung cancer (SCLC) and large cell neuroendocrine carcinoma (LCNEC) (200, 201).

PTPN6 shows a negative regulatory effect on the activation of T cells, natural killer (NK) cells and macrophages. However, deletion of PTPN6 significantly strengthens the capacity of these immune cells for tumor killing and promotes anti-tumor immunity (202–204). Of note, a considerable and durable T cell-mediated suppression of tumor growth was observed when PTPN6 knockdown of OT-I T cells was combined with *anti*-PD-1 and cytotoxic T-lymphocyte-associated protein 4 (CTLA-4) immunotherapy (205). And the ability of tumor-associated DCs (TADCs) and MoDCs to take up and process immune complexes (IC) containing tumor antigens bound to antitumor antibody, ultimately inducing anti-tumor immunity *in vivo*, was augmented by simultaneous inhibition of PTPN6 and phosphatases regulating AKT activation (206).

PTPN11, involved in the regulation of tumor and immune cell signaling, is a critical modulator of PD-1 and B and T lymphocyte attenuator (BTLA) immune checkpoint pathways and promising drug target in tumor immunotherapy (207). Inhibition of PTPN11 activity enhances tumor-intrinsic IFN-γ signaling, resulting in increased chemoattractant cytokine release and cytotoxic T cell recruitment, as well as increased expression of major histocompatibility complex (MHC) class I and programmed cell death ligand 1 (PD-L1) on the surface of cancer cells, along with decreased differentiation and suppression of immunosuppressive myeloid cells in the tumor microenvironment (208, 209). In highly aggressive mouse models of breast cancer and melanoma, simultaneous suppression of CSF-1R and PTPN11 to activate macrophages and promote phagocytosis may be an effective strategy for macrophage-based immunotherapy (210). Mechanistically, PTPN11 deletion attenuates CSF-1 receptor signaling, which depletes pro-tumor M2 macrophages while increasing anti-tumor M1 macrophages (211). On the other hand, deletion of PTPN11 enhances macrophage response to IFN-γ and increases production of the tumor cell-derived cytokine CXCL9, thereby promoting tumor infiltration of IFN-γ-producing T cells (212). More importantly, PTPN11 inhibitors combined with immunotherapies, such as *anti*-PD-1/L1, would reverse immunosuppression in the tumor microenvironment (TME) and potentiate the systemic antitumor effect in NSCLC cancer (213, 214).

Currently, research on PTPN22 is not only limited to autoimmune diseases, but more evidence indicates a considerable importance in tumor immunity. Implantation of syngeneic tumors in PTPN22^-/-^ mice resulted in greater infiltration and activation of macrophages, NK cells and T cells, which in turn led to spontaneous tumor regression. More importantly, the combination with anti-PD-1/L1 therapy in the presence of PTPN22 inhibition significantly enhanced the antitumor efficacy (215, 216).

Based on the above findings, PTPN family members still serve as negative regulators in the immune system by restricting the development and differentiation of immune cells to achieve anti-inflammatory and anti-autoimmune responses. But on the other hand, PTPNs could make tumor cells evade immune surveillance.

## Non-coding RNAs regulate the role of PTPNs in cancers

**Figure 4:**
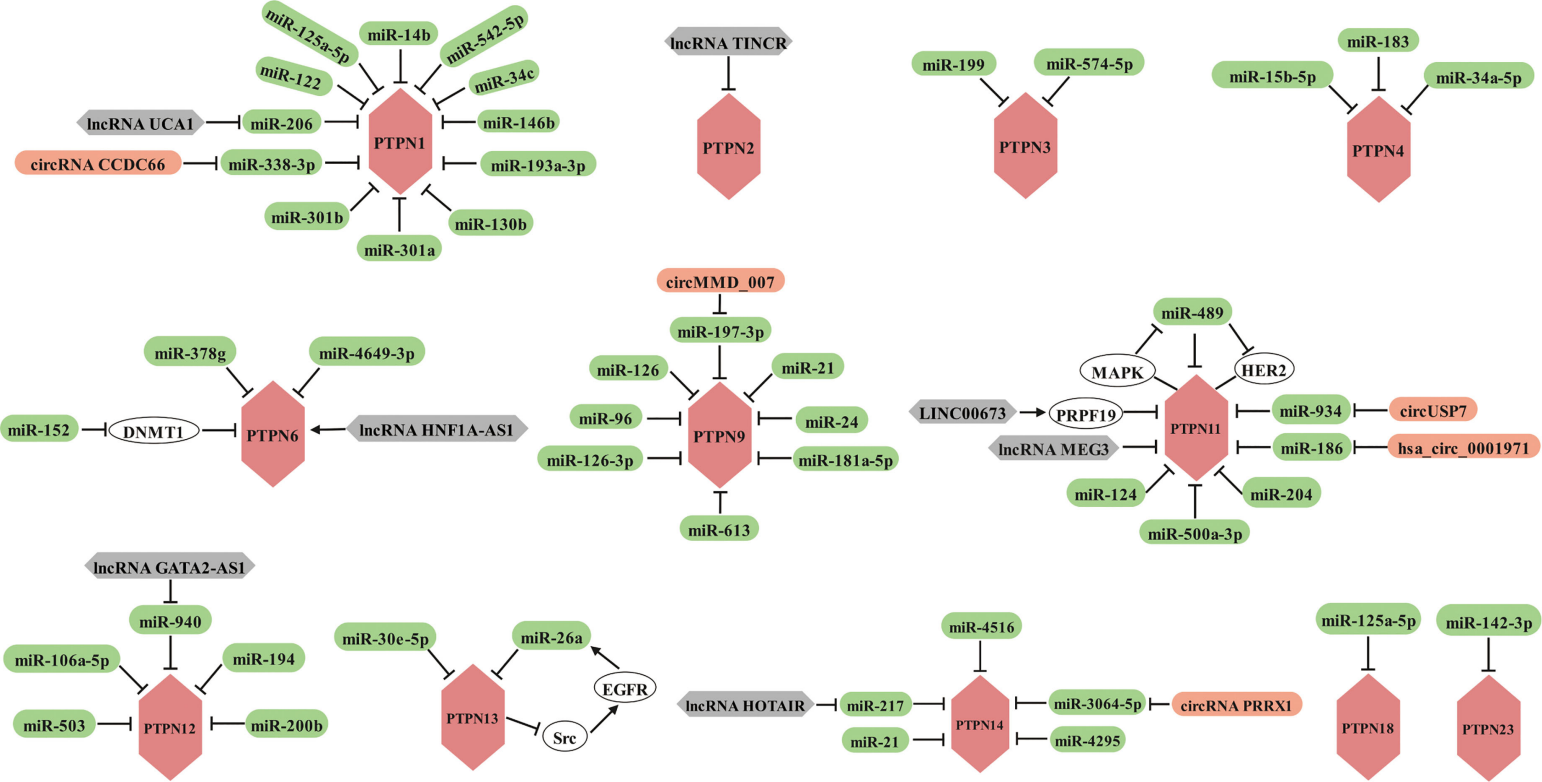
Members of the PTPN family are regulated by miRNAs, lncRNAs and circRNAs in various cancers.

A substantial body of evidence has demonstrated that inhibition of some PTPN family members can considerably improve the efficacy of antitumor immunity, and the availability of small molecule inhibitors has given hope. However, drug discovery is an extremely long process, so the search for new therapeutic tools is urgent. The presence of a large number of non-protein-coding RNAs in the human genome and the potential for these non-coding RNAs to affect normal gene expression and disease progression make them a new class of targets for drug discovery (217). Therefore, we present here a comprehensive summary of ncRNAs, including miRNAs, lncRNAs and circRNAs, involved in the regulation of PTPNs in tumors and other diseases (**Figure 4**).

### MicroRNAs are implicated in the regulation of tumorigenesis, progression, metastasis, and drug resistance by targeting PTPNs

Currently, it is widely reported that miRNAs can influence the disease process, especially in cancer, by regulating the expression of PTPN1. In hepatocellular carcinoma, miR-122 and miR-206 target the 3’ untranslated region (3’ UTR) of PTPN1 mRNA and induce its degradation (218, 219), while miR-125a-5p suppresses PTPN1 expression *via* the MAPK signaling pathway (220), both of which ultimately alleviated the progression of hepatocellular carcinoma. Interestingly, miR-14b reverses the EMT phenotype of cisplatin-resistant lung adenocarcinoma cells by targeting PTPN1 (221). PTPN1 has also been discovered to be a target of numerous miRNAs in various malignancies. PTPN1 is targeted by miR-542-5p and miR-34c in glioma (222, 223), miR-146b and miR-338-3p in gastric cancer (224, 225), and miR-193a-3p in breast cancer (226). By regulating PTPN1, the miRNAs described above serve as tumor suppressors in cancers. Conversely, by targeting PTPN1, a tumor suppressor in bladder cancer, the miR-130 family (miR-130b, miR-301a, and miR-301b) contributes to cancer development (127).

The previously mentioned PTPN3 can confer chemotherapy resistance and tumor stem cell-like characteristics to ovarian cancer cells, but its expression is regulated by miR-199 (82). What’s more, miR-574-5p facilitates phosphorylation of p44/42 MAPKs by targeting PTPN3, thereby promoting angiogenesis in gastric cancer (227).

PTPN4 appears to function as a tumor suppressor in cancer, miR-15b-5p activates STAT3 signaling by targeting PTPN4 to promote oral squamous cell carcinoma progression (228). And miR-183 promotes migration and invasion of CD133^+^/CD326^+^ lung adenocarcinoma initiating cells by inhibiting PTPN4 (229). Furthermore, miR-34a-5p inhibits ROS-mediated apoptosis in papillary thyroid cancer cells through down-regulation of PTPN4 (230).

The regulation of PTPN6 is primarily mediated by a tiny proportion of miRNAs. Among them, miR-152 indirectly promotes PTPN6 expression to suppress lymphoma growth through down-regulation of DNA methyltransferase 1 (DNMT1) (231). But in nasopharyngeal carcinoma, miR-4649-3p inhibits cell proliferation and miR-378g partially enhances the radiosensitivity of NPC cells both by targeting PTPN6 (232, 233).

PTPN9 exhibits various functions depending on the type of tumor. PTPN9 promotes proliferation and invasion of esophageal cancer cells and non-small cell lung cancer and is negatively regulated by miR-126 and miR-126-3p, respectively (234, 235). But in cervical, breast, colorectal and gastric cancers, PTPN9 functions as a tumor suppressor with an essential role in suppressing tumor proliferation, invasion and migration. However, PTPN9 is targeted by miR-613 and miR-96 in cervical cancer (236–238), miR-96 and miR-24 in breast cancer (239, 240), miR-21 in colorectal cancer (241), and miR-181a-5p in gastric cancer (242).

PTPN11, which is considered an oncogenic factor and a key target for cancer immunotherapy according to several studies, is mediated by several miRNAs. MiR-124 and miR-489 inhibited the progression of renal cell carcinoma and hypopharyngeal squamous cell carcinoma by suppressing the expression of PTPN11, respectively (243, 244). In cutaneous squamous cell carcinoma, miR-204 inhibits STAT3 and facilitates the MAPK signaling pathway, possibly through PTPN11, a direct target of miR-204 (245). In oral squamous cell carcinoma, miR-186 directly binds to 3’ UTR of PTPN11 mRNA and inhibits the expression, which suppresses the signaling activity of Erk and AKT that is required for cancer cell growth (246). In hepatocellular carcinoma, miR-186 inhibits self-renewal of hepatocellular carcinoma stem cells and is more sensitive to cisplatin treatment by binding to 3’-UTR of PTPN11 mRNA and reducing its expression (247). However, miR-500a-3p promotes HCC cancer stem cell properties by targeting PTPN11, a negative regulator of the JAK/STAT3 signaling pathway (248). Moreover, HER2 is a direct target of miR-489, overexpression of miR-489 suppresses breast cancer invasion by attenuating HER2-PTPN11-MAPK signaling, which in turn inhibits miR-489, producing a mutually inhibitory loop (249).

PTPN12 suppresses progression of multiple cancers and is negatively controlled by miR-106a-5p in hepatocellular carcinoma (250), miR-503 in retinoblastoma (251), miR-194 in ovarian cancer (252), and miR-200b in colon cancer (253).

MiR-30e-5p promotes lung adenocarcinoma cell growth by targeting PTPN13 and is detrimental to the survival of LUAD patients (254). What’s more, miR-26a desensitizes NSCLC cells to tyrosine kinase inhibitors by targeting PTPN13. Mechanistically, miR-26a, which is downstream of EGFR signaling, directly targets and silences PTPN13 to maintain activation of Src, the dephosphorylated substrate of PTPN13, thereby enhancing the EGFR pathway in regulatory circuits (255).

By targeting PTPN14, miR-21 promotes intrahepatic cholangiocarcinoma proliferation and growth *in vitro* and *in vivo* (256), miR-4295 and miR-4516 contribute to the progression of osteosarcoma and glioblastoma, respectively (257, 258). But miR-217 inhibits EMT in gastric cancer (259).

In addition, miR-125a-5p is implicated in imatinib resistance in gastrointestinal stromal tumor (GIST). Mechanistically, overexpression of miR-125a-5p suppresses PTPN18 expression and subsequently enhances phosphorylated FAK (pFAK) expression in GIST cells, which contributes to imatinib resistance in GIST (260, 261).

PTPN23, which features a tumor suppressor function in testicular germ cell tumors, is regulated by miR-142-3p (262).

In light of the fact that almost all miRNAs target and suppress the expression of PTPNs, miRNA modulation combined with immunotherapy may be a novel therapeutic strategy.

### LncRNAs and circRNAs regulate the role of PTPNs in cancers in part by sponging miRNAs

In recent years, a growing review of the literature revealed that lncRNA and circRNA appear to play a pivotal role in cancer and hold considerable promise as novel biomarkers and therapeutic targets (263). Next, we illustrate the mechanisms by which lncRNAs and circRNAs modulate PTPN family members, respectively.

PTPN family members have been shown to be regulated by different lncRNAs, including but not limited to lncRNA UCA1, TINCR, HNF1A-AS1, LINC00673, MEG3, GATA2-AS1, and HOTAIR. Specifically, lncRNA UCA1 accelerates the proliferation of breast cancer cells based on the miR-206/PTPN1 axis (264). In hepatocellular carcinoma, lncRNA TINCR interacts directly with and inhibits PTPN2 to promote the proliferation and invasion through activating STAT3 signaling (265). However, lncRNA HNF1A-AS1 reverses the malignancy of hepatocellular carcinoma by enhancing the phosphatase activity of PTPN6 (266). Furthermore, LINC00673 can strengthen the interaction of PTPN11 with PRPF19, an E3 ubiquitin ligase, and promote PTPN11 degradation through ubiquitination, resulting in reduced Src-Erk oncogenic signaling and enhanced STAT1-dependent antitumor response activation (267). PTPN11 can be targeted by lncRNA MEG3, thereby suppressing the proliferation and metastasis of renal cell carcinoma (243). On the basis of the miR-940/PTPN12 axis, lncRNA GATA2-AS1 restrains esophageal squamous cell carcinoma progression (268). In contrast, ectopic expression of lncRNA HOTAIR promotes drug resistance and augments GC cell proliferation and migration by inhibiting miR-217 expression and enhancing PTPN14 expression (269).

CircRNAs with similar regulatory mechanisms to lncRNAs are implicated in the regulation of PTPN family members, namely circRNA CCDC66, circMMD 007, circUSP7, has_circ_0001971, and circPRRX1. CircRNA CCDC66, in particular, promotes osteosarcoma proliferation and metastasis by sponging miR-338-3p to increase PTPN1 expression (270). Likewise, circMMD_007 promotes oncogenic effects in lung adenocarcinoma progression *via* the miR-197-3p/PTPN9 axis (271). CircUSP7 renders NSCLC patients resistant to *anti*-PD1 immunotherapy. Mechanistically, circUSP7 suppresses CD8^+^ T cell function by sponging miR-934 to up-regulate PTPN11 expression (272). Moreover, hsa_circ_0001971 promotes the proliferation of oral squamous carcinoma cells *via* miR-186/PTPN11 axis (273). Finally, circPRRX1 strengthens doxorubicin resistance in gastric cancer by regulating miR-3064-5p/PTPN14 signaling (274).

Taken together, lncRNAs and circRNAs serve as oncogenic or tumor suppressors and regulate the expression of PTPNs overwhelmingly through sponging miRNA, thereby dominating cancer progression.

## Conclusion and perspective

Over the last three decades, a growing series of investigations have been able to conclude that PTPNs perform an essential role in almost all phenotypes of tumor cells. As described previously, there are numerous members of the PTPN family that all exert distinct functions in various malignancies, although they share the same catalytic structural domain. Some PTPNs exploit their structural domains with phosphatase activity to dephosphorylate a variety of oncogenic substrate proteins to achieve activation or inactivation, thereby participating in the regulation of cancer progression. Importantly, some members confer stem cell-like and EMT characteristics to tumor cells. The functions and signaling pathways regulated by PTPN family members are tissue- and cell-specific, so most PTPNs exert their functions still depend on the type of tumor in which they are located, but a small proportion of members still serve more specific functions, for instance, PTPN11 exerts oncogenic effects, while PTPN13 holds a tumor suppressive role in almost all cancers. Currently, although part of the mechanisms underlying the engagement of PTPN family members in tumors have been elucidated, the understanding of their operational mechanisms is even more challenging and urgent. In the future, a better understanding of the multifunctional and sophisticated regulatory mechanisms of PTPNs may be significant for the development of more specific targeted therapeutic strategies.

What’s more, PTPNs also hold critical role in cancer treatment, growing evidence suggested that PTPNs are implicated in chemotherapy and radiotherapy resistance. For instance, PTPN11 confers cisplatin resistance in small cell lung cancer, while PTPN6 promotes radiation resistance in nasopharyngeal carcinoma. Furthermore, inhibiting PTPNs, regardless of immune checkpoint inhibitors or CAR T therapies, can significantly improve the efficacy of immunotherapy. Currently, PTPN11 is the most reported to be associated with tumor immunotherapy and has spawned multiple inhibitors, including but not limited to SHP099 and TN0155, which in combination with *anti*-PD-1 and *anti*-PD-L1 therapies can significantly improve the malignant characteristics of tumors (209, 213, 275). Accordingly, further development of novel PTPNs inhibitors and investigation of the safety of these compounds is urgently need for conquering cancer in future.

Given the special role of PTPNs in cancer progression and immunotherapy, the question about how PTPNs inhibitors can move from the laboratory to rational clinical application should be the next thing to consider. As previously described, PTPNs perform a negative regulatory role in the immune system. Inhibition of PTPNs can activate a variety of immune cells involved in the killing of tumor cells, and also significantly potentiate the efficacy of cancer immunotherapy. In brief, PTPN inhibitors are positive modulators of antitumor immunotherapy. Then regarding the rational use of PTPNs inhibitors there are three situations: first, if PTPN plays a carcinogenic role, the usage of PTPN inhibitors will dramatically enhance the antitumor efficacy by suppressing the carcinogenic properties of PTPN while activating the immune system to combat tumors. Second, if PTPN exerts a tumor suppressive function, the use of PTPN inhibitors needs to be more cautious because it is not clear whether the anti-cancer effect of PTPN itself or the anti-tumor immune effect of PTPN inhibition is stronger, which needs to be further studied in mouse models. Third, there is the issue of the dual-sidedness of PTPN in specific cancers that we mentioned earlier, which requires even more *in vivo* experiments to further explore. Therefore, the rational and accurate clinical use of these inhibitors depends on the individual situation.

As we all know, the inhibitors applied in the clinic must be with high specificity. For the development of PTPN inhibitors, there is still a thorny issue of specificity. Furthermore, designing drug-like inhibitors for PTPNs is challenging due to their highly conserved and positively charged active site structures (276). Hence, the elucidation of the mechanisms regulating PTPNs may become a new therapeutic strategy. Here, we systematically summarize the ncRNAs involved in the regulation of PTPNs as an alternative to developing new inhibitors and propose that ncRNAs, especially miRNAs, in combination with immunotherapy, which may be a promising therapeutic approach to combat tumors.

## Author Contributions

XT and CQ participated in consolidation of information and writing. HZ and YL guided and supervised this study. All authors contributed to the article and approved the submitted version.

## Funding

This work was supported by National Natural Science Foundations of China (81802400, 81902519) and China Postdoctoral Science Foundation (2020M670053). This work was also supported in part by the Postdoctoral Fellowship of Peking-Tsinghua Center for Life Sciences.

## Conflict of Interest

The authors declare that the research was conducted in the absence of any commercial or financial relationships that could be construed as a potential conflict of interest.

## Publisher’s Note

All claims expressed in this article are solely those of the authors and do not necessarily represent those of their affiliated organizations, or those of the publisher, the editors and the reviewers. Any product that may be evaluated in this article, or claim that may be made by its manufacturer, is not guaranteed or endorsed by the publisher.
